# *Plectranthus amboinicus*: A Systematic Review of Traditional Uses, Phytochemical Properties, and Therapeutic Applications

**DOI:** 10.3390/ph18050707

**Published:** 2025-05-10

**Authors:** Márcia Santos Filipe, Gabrielle Bangay, Florencia Z. Brauning, Festus Oladayo Ogungbemiro, Bernardo Brito Palma, Ana María Díaz-Lanza, Amr Hassan, Rebeca André, Patricia Rijo

**Affiliations:** 1 CBIOS—Research Center for Biosciences & Health Technologies CBIOS, Universidade Lusófona, Campo Grande 376, 1749-024 Lisboa, Portugal; marcia.filipe@ulusofona.pt (M.S.F.); gabrielle.bangay@ulusofona.pt (G.B.); fzbrauning@gmail.com (F.Z.B.); bernardo.palma@ulusofona.pt (B.B.P.); rebeca.andre@ulusofona.pt (R.A.); 2 Departamento de Ciencias Biomédicas—Área de Farmacología, Nuevos Agentes Antitumorales, Acción Tóxica Sobre Células Leucémicas, Facultad de Farmacia, Universidad de Alcalá de Henares, Ctra. Madrid-Barcelona km. 33,600, 28805 Alcalá de Henares, Madrid, Spain; ana.diaz@uah.es; 3 Department of Chemistry, Federal University of Lafia, Lafia 950101, Nigeria; festusoladayoonline@gmail.com; 4 Computational and Bio-Simulation Research Group, Department of Chemistry, University of Calabar, Calabar 540281, Nigeria; 5 Department of Bioinformatics, Genetic Engineering and Biotechnology Research Institute (GEBRI), University of Sadat City, Sadat 32897, Egypt; amrhassan.nanotechnology@gmail.com; 6 Instituto de Investigação do Medicamento (iMed.ULisboa), Faculdade de Farmácia, Universidade de Lisboa, 1649-003 Lisboa, Portugal

**Keywords:** *Plectranthus amboinicus*, skin, phytochemistry, traditional uses, antimicrobial activity, *Coleus amboinicus*, *Lamiaceae*

## Abstract

**Background:** *Coleus amboinicus* (Lour.) (syn *Plectranthus amboinicus*) from the Lamiaceae family—a large family of aromatic herbs with many medicinally important species—is a frequently cited species within the *Plectranthus* genus, renowned for its traditional uses, phytochemical composition, biological activities, and applications in skin care. **Methods**: A systematic review was conducted following PRISMA guidelines to provide an in-depth understanding of *P. amboinicus*’ phytochemical composition and biological activity, particularly in dermatological contexts, underscoring its significance in traditional medicine and modern phytochemical research. **Results**: *P. amboinicus* extracts and essential oils exhibit significant antimicrobial activity against both Gram-positive and Gram-negative bacteria, and notable antifungal properties, particularly against dermatophytes. Additionally, the species demonstrates remarkable mosquito repellent and anti-parasitic effects, comparable to DEET, and potent anti-inflammatory properties by inhibiting pro-inflammatory cytokines. The plant’s rich polyphenolic content contributes to its significant antioxidant properties, preventing conditions like hyperpigmentation and premature aging. *P. amboinicus* also exhibits cytotoxic activity against various cancer cell lines and promotes wound healing through its analgesic, anti-inflammatory, and antioxidant abilities. **Conclusions**: This comprehensive exploration of *P. amboinicus* validates its diverse therapeutic potential across infectious diseases, oncology, and wound care. Further research and clinical trials are warranted to fully elucidate its mechanisms of action and optimize its therapeutic applications, paving the way for its integration into mainstream medical practices.

## 1. Introduction

### 1.1. The Skin

The skin, the outermost human organ, is vital for maintaining overall health and homeostasis [1]. Its role as a primary defense barrier protects against environmental threats, such as ultraviolet (UV) radiation, physical and chemical injuries, infections, and water loss [2]. The proper functioning and appearance of the skin are maintained by a delicate balance between the water content of the stratum corneum and skin surface lipids [3].

The skin is composed of three primary layers: the epidermis, dermis, and subcutaneous tissue [2]. These structures play a critical role in preventing the invasion of microorganisms or pathogens, protecting the body from physiological damage and preventing excessive moisture loss. These protective functions are collectively known as “barrier functions”. Furthermore, the skin undergoes oxidative damage due to various stresses, including daily exposure to UV rays from the sun. This exposure leads to the generation of reactive oxygen species (ROS), such as singlet oxygen and secondary lipid peroxyl radicals. These radicals damage biological molecules, including proteins and DNA, disturbing healthy skin conditions [4].

Given all these considerations, it is crucial to take care of the skin, the largest human organ, and pay attention to all the signs it gives. Proper skincare is essential to maintaining its protective functions and overall health.

### 1.2. Traditional Herbal Medicine

Since ancient times, people have relied on traditional herbal medicines for healing [5]. Ancient civilizations, including the Egyptians, Indians, and Chinese, used local plants to treat various diseases since at least 2500 BC. Through generations of trial and error, a system of medicine evolved incorporating unique formulations based on plant parts. According to the World Health Organization (W.H.O), 80% of the population in developing countries still depend on traditional medicines, mostly derived from plants [6,7].

In recent years, there has been a global resurgence of interest in the traditional medical system. Screening of medicinal herbs has become a promising source of bioactive compounds with therapeutic value, driving phytochemical research. Ethnomedicobotanical study of tribal communities can reveal efficient herbal drugs [5]. The art of herbal healing is deeply rooted in many cultures and folklores. Reliance on herbal medicine is even greater among people living in remote forests, such as the Western Ghats in India, where access to modern medical facilities is limited [8]. In Singapore, the local Chinese use Traditional Chinese Medicine (TCM), the Malays use Jamu Medicine, and the Indians practice Ayurvedic, Unani, and Siddha Medicine. These herbs are typically consumed in dried forms or as finished products, but some locals still use fresh medicinal plants. The herbal knowledge in these regions has been passed down verbally from older generations [9,10].

Skin infections are often caused by the microorganisms that invade the skin tissues. Herbal plants are valuable sources of diverse compounds, including phenolic compounds, terpenoids, vitamins, and secondary metabolites, which possess antioxidant, antimicrobial, anti-inflammatory, antitumor, anti-mutagenic, anti-carcinogenic, and diuretic activities [7]. Medicinal plants are recognized for their potential as natural antioxidants and are widely used in wound healing [11]. The discovery of active compounds in individual plants and their specific biological actions allows for the treatment of various pathologies. Herbal preparations often involve multiple species and plant parts, enhancing the therapeutic efficacy through chemical interactions and increased treatment effectiveness [12].

In ethnobotany, plant mixtures are commonly used, though their compositions are not always documented as thoroughly as individual plant therapies. These mixtures, chosen systematically for their therapeutic potential, often include leaves, stems, barks, roots, rhizomes, bulbs, and seeds. The complexity of these mixtures usually depends on the severity of the illness being treated. Simple preparations may suffice for minor ailments, while more elaborate mixtures are necessary for severe conditions [12]. Aromatic plants and their essential oils are significant sources of active compounds used in various applications, medicinally important vectors, and agricultural pest control. Recent studies have demonstrated the bioactivity of aromatic plant extracts and essential oils, highlighting their potential in both medical and agricultural contexts [13].

Overall, the therapeutic efficacy of combining medicinal species can be enhanced with the right combinations, promoting synergism or potentiating therapeutic effects. However, understanding the possible negative effects is crucial, as incorrect combinations can be harmful and drug–drug interactions can occur. The growing interest in traditional herbal medicine underscores the need for continued research and documentation to preserve this valuable knowledge and ensure its safe and effective use.

### 1.3. Plectranthus Genus

The Lamiaceae family is renowned for its economic and medicinal importance, encompassing around 4000 species globally [14]. This family has been extensively studied across various disciplines, highlighting its significant ethnobotanical benefits [15,16]. Notable genera within the Lamiaceae family include *Plectranthus*, *Salvia*, *Ocimum,* and *Mentha*, each recognized for their therapeutic, nutritional, and horticultural properties.

Historically, *Plectranthus* was a large and heterogeneous genus within the Lamiaceae family, comprising around 300 species, primarily distributed across warm regions of Africa, Asia, and South America [17,18]. Over 85% of the literature on *Plectranthus* focuses on its therapeutic properties, followed by its nutritional and horticultural applications- largely attributed to its aromatic and essential oil production [19].

The name *Plectranthus* derives from the Greek words “plectron”, meaning spur, and “Anthos”, meaning flower, referring to the spur-shaped flowers of some species within the genus [20]. Due to the lack of precise morphological features for distinguishing species within *Plectranthus* and its closely related genera, numerous taxonomic challenges have arisen, leading to the misplacement of species into closely linked genera such as *Coleus*, *Solenostemon,* and *Englerastrum* [19,20]. As a result, several species previously assigned to *Plectranthus*, including *Plectranthus amboinicus*, have been reclassified under the resurrected genus *Coleus*.

Species of *Plectranthus* are notable for their biosynthetic production of sesquiterpenes, diterpenes, and phenolic compounds, many of which possess significant biological properties [21]. These species are recognized for their antimicrobial, anticancer, anti-parasitic, repellent, immunomodulating activity, among others [22]. The bioactivities of *Plectranthus* are largely attributed to its rich content of phenolic compounds, including flavonoids (flavones, flavonols, and flavonones) and phenolic acids, such as trans-rosmarinic acid [23,24,25,26,27]. These compounds contribute to the genus’ therapeutic potential and its economical and medicinal value [19].

### 1.4. Plectranthus amboinicus

*Plectranthus amboinicus* (Lour.) Spreng, a synonym for the currently accepted taxonomical name *Coleus amboinicus* Lour., is a fleshy, succulent herb recognized for its distinct oregano-like flavor and aroma [19]. There is no certainty of the origin of this plant, but it is believed that it comes from Africa and India [28]. Nowadays, *P. amboinicus* is widely distributed throughout tropical and warm regions of Africa, Asia, Australia, and the Americas [29,30].

*P. amboinicus* belongs to *Plectranthus* genus, within the Lamiaceae family, but it was initially classified under the genus *Coleus*, which is why both names are still commonly used in the literature. As a result, there are several synonym names for this plant, such as *P. aromaticus* Roxb., *Coleus aromaticus* Benth., and *C. amboinicus* Lour [19]. Different populations know this plant by different names, such as country borage (England), Indian borage, Cuban oregano, French thyme, Spanish thyme, Mexican mint, soup mint, Bangun-bangun (Malaysia), Ajwain patta (Hindi), Karpuravalli (Tamil), Pani Koorka (Malayalam), and Malvarisco, Malvariço, or Hortelã-graúda (Brazil) [7,31,32].

### 1.5. Morphology of P. amboinicus

*P. amboinicus* is a large succulent aromatic herb, perennial with a 3–10-year lifespan [33]. The leaves of *P. amboinicus* are thick, fleshy, and broadly ovate with a highly aromatic scent, due to glandular hairs on the lower surface [28]. Additionally, the leaves are green, pubescent, with crenate margins, are heart-shaped, and grow opposite each other on petioles [34,35].

*P. amboinicus* stems are fleshy, flexible, and can grow to 30–90 cm long, covered with either long rigid hairs (hispidly villous) or dense short hairs (tomentose) [19]. *P. amboinicus* has a fibrous structure with many nodes and internodes. *P. amboinicus* flowers are pale purplish, bell-shaped, and arranged in dense whorls on a long slender raceme. They have a smooth throat with two lips, the upper lip being ovate and thin and the lower lip having four narrow teeth [36].

### 1.6. Applications and General Uses of P. amboinicus

*P. amboinicus* presents a distinctive oregano-like and refreshing odor. Due to this unique characteristic, this plant is often used in culinary practices to enhance flavor and aroma [35,37]. It is commonly prepared as an infusion, decoction, or syrup in traditional cuisines and remedies [38]. In terms of propagation, *P. amboinicus* rarely produces viable seeds and is typically propagated through stem cuttings, which root easily and support its fast-growing nature [19].

Behind its culinary use, *P. amboinicus* is an important plant in traditional medicine, in several countries, for various illnesses. The use of this plant depends on cultural customs and the purpose of its application, with dry and fresh leaves being widely used in the preparation of infusions, decoctions, soups, and syrups [39]. Furthermore, direct topical use of fresh leaves has been widely reported, mainly to treat skin problems such as burns, bruises, insect bites, and skin inflammation [40].

Based on the traditional knowledge across several countries, *P. amboinicus* is used to treat a wide variety of ailments. In India, the leaf juice is commonly applied for respiratory issues such as coughs, sore throats, nasal congestion, asthma, and bronchitis [41,42]. In Brazil, it is used for digestive problems and for the treatment of skin ulcerations caused by *Leishmania braziliensis* [18]. In Southeast Asia, the plant is traditionally used to treat some skin conditions. Across South America and the Caribbean, it is used topically to treat skin conditions such as inflammations, abrasions, lacerations, cutaneous leishmaniasis, and hard-to-heal wounds [5,8,43,44]. It is also used for digestive problems such as colic, indigestion, constipation, flatulence, dyspepsia, diarrhea, cholera, colitis, and liver conditions (hepatopathy) [42,45]. In African traditional medicine, *P. amboinicus* is used for microbial infections, fever, and helminthiasis [38,42,46]. The plant is used in Indian and Nigerian folk medicine for neurological conditions such as convulsions, epilepsy, and headaches [47,48]. Its anti-inflammatory and analgesic properties are recognized in traditional practices in Indonesia and West Africa, where it is used for arthritic pain and general inflammations [36,38,49]. The plant is also employed in China and Nigeria to address urinary and renal issues, including kidney and bladder stones, and painful urination (strangury) [32,47]. In tropical countries, it is traditionally used for treating malarial fever [47]. Other uses include its application as an anthelmintic, a bronchodilator, a remedy for animal and insect bites, and a lactagogue to promote breast milk production [40,50,51,52].

In recent years, different therapeutic activities have been scientifically proven in different experimental models, such as antimicrobial [53,54], anti-inflammatory [38,55,56,57], antitumor [56,58,59], anti-diabetic [60], diuretic, anti-epileptic [30], anti-viral [61,62], antibacterial [41,45], antifungal (fungicidal and fungistatic) [31], anti-larvicidal [13,33], anxiolytic [63], and wound healing [49,50]. The therapeutic properties of *P. amboinicus* have been directly related to the phytochemical composition of the plant, which is rich in bioactive compounds, particularly in terms of flavonoids and cinnamic derivative compounds [12,63,64].

Based on *P. amboinicus*’ reported uses and potential therapeutic applications, this review aims to offer an in-depth understanding of its phytochemical composition and biological activities, particularly in dermatological contexts, validating its traditional uses and outlining its significance in modern phytochemical research.

## 2. Results and Discussion

### 2.1. Phytochemistry

Phytochemicals that are considered as primary metabolites play a vital role in the growth, development, or reproduction of a plant. Alternatively, secondary metabolites are plant compounds considered to be involved in non-essential processes, such as a plant’s defense system and pollination. Many classes of these secondary metabolites have been identified in *P. amboinicus*. In particular, monoterpenoids, diterpenoids, triterpenoids, sesquiterpenoids, alkaloids, phenolics, and flavonoids have been reported [19,29,65], with one study even suggesting that *P. amboinicus* contains 76 volatile and 30 non-volatile compounds [19].

#### 2.1.1. Leaves

The phenomenon that different parts of the same plant can contain differing concentrations of phytochemicals is well studied. In one study on *P. amboinicus*, the presence of triterpenoids and saponins was found to be higher in the root extract compared to the leaf or stem [36]. Many of the studies carried out on *P. amboinicus* focus on the monoterpenoids and other volatile compounds isolated from the essential oil of the plant. However, this section addresses studies that have examined the leaf extract for non-volatile compounds.

In general, alkaloids, tannins, triterpenoids, saponins, carotenoids phenolics, sesquiterpenes, phenolic acids, and flavonoids (flavones, flavonones, anthocyanidins, xanthones, chalcones, aurones) have been identified from the leaf extract of *P. amboinicus* [7,36,47,66,67,68,69]. In particular, flavonoids, such as luteolin, quercetin, agpigenin, cirsimartin, salvigenin, rutin, luteolin, genkwanin [36,47], and hydroxycinnamic acids, including chlorogenic, caffeic, coumaric, and rosmarinic acid [45], have been documented. Contrary to expectations, some phytochemical analyses of *P. amboinicus* extracts did not detect anthocyanidins, suggesting variability influenced by environmental factors like drought. In one study, HPLC/DAD identified 10 compounds in the fresh leaf extract of *P. amboinicus*, including gallic acid, catechin, chlorogenic acid, caffeic acid, rutin, quercitrin, isoquercitrin, quercetin, kaempferol, and glycosylated kaempferol [22]. One investigation identified, for the first time, 16 pure compounds (out of 21), including flavonoids from the methanol extract of *P. amboinicus* after the leaves were subjected to chromatographic separation. The compounds included 5-hydroxy-7,4′-dimethoxyflavone (1), 5-hydroxy-7,3′,4′-trimethoxyflavone (2), 5,3′-dihydroxy-7,4′-dimethoxyflavone (3), 5,4′-dihydroxy-7,3′-dimethoxyflavone (4), 5,4′-dihydroxy-7-methoxyflavone (5), 3,5-dihydroxy-7,3′,4′,5′-tetramethoxyflavone (6), 5,8-dihydroxy-7,3′,4′-trimethoxyflavone (7), 5,8-dihydroxy-7,4′-dimethoxyflavone (8), 5,8-dihydroxy-7,2′,3′,5′-tetramethoxyflavone (9), 5-hydroxy-7,2′,3′,5′-tetramethoxyflavone (10), 5-hydroxy-7,2′,3′,4′,5′,6′-hexamethoxyflavanone (11), 4-hydroxy-4-methylpentan-2-one (diacetone alcohol) (12), linoleic acid (13), 7-oxooctanoic acid (14), vanillic acid (15), *p*-hydroxybenzoic acid (16), *p*-hydroxybenzaldehyde (17), *o*-hydroxybenzaldehyde (18), pheophytin a (19), pheophytin b (20), and aristophyll-C (21) [29].

A study on *P. amboinicus* leaves from Egypt isolated eight compounds from the ethyl acetate fraction, verified by NMR. These compounds included 3-methoxy genkwanin, crisimaritin, p-coumaric acid, caffeic acid, taxifolin, rosmarinic acid, apigenin, and 5-O-methyl-luteolin. Notably, 3-methoxy genkwanin, p-coumaric acid, and 5-O-methyl-luteolin were reported in this plant for the first time. The total phenolic content was higher in the stem extract (9.6 mg/g) compared to the leaf (8.4 mg/g) and root extracts (5.4 mg/g) and the tannin content was highest in the root extracts (126 µg/g), followed by the leaf (90 µg/g) and stem extracts (81 µg/g). The chemical composition of aqueous leaf extracts included tannins, flavonoids, saponins, polyuronides, and steroid glycosides [19].

Crucially, the solvent and method used in the extraction process has a determinant outcome on the compounds isolated from the plant. Solvents, such as ethanol, methanol, and water lead to the isolation of tannins from *P. amboinicus* [7]. In another study, rosmarinic acid, ladanein, and cirsimaritin were found in both the methanolic and aqueous leaf extracts of *P. amboinicus* [65], whereas the ethanolic extract from fresh leaves of *P. amboinicus* contained the cytotoxic diterpene 7-acetoxy-6-hydroxyroyleanone, verified using UPLC-QTOF-MS/MS [59]. Another investigation into the methanolic extract of *P. amboinicus* leaves yielded 12 pure substances, including 4 flavonoids (5-hydroxy-7,4′-dimethoxyflavone, 5-hydroxy-7,3′,4′-trimethoxyflavone, 5,3′-dihydroxy-7,4′-dimethoxyflavone, and 5,4′-dihydroxy-7,3′-dimethoxyflavone); three benzenoids (vanillic acid, p-hydroxybenzoic acid, and methylparaben); one quinol (4-acetonyl-3,5-dimethoxy-p-quinol); three steroids (β-sitosterol, β-sitostenone, and stigmastenone); and one lignan ((+)-syringaresinol), having been isolated from *P. amboinicus* leaves for the first time [70]. The preliminary screening of *P. amboinicus* leaves identified quinones, flavonoids, phenols, and terpenoids present in small amounts only in the ethanolic extract, while the chloroform and aqueous extracts showed minimal presence of these compounds. Moreover, the ethanolic extract contained significant amounts of total flavonoids, alkaloids, and phenolic content, measuring 25.65 ± 1.5 mg/g, 18.27 ± 1.36 mg/g, and 10.15 ± 0.36 mg/g, respectively, comparable to the chloroform and aqueous extracts [71]. In another study, the authors identified the presence of rosmarinic (0.0573 mg/g), caffeic (0.056 mg/g), *p*-coumaric acid (0.0746 mg/g), and quercetin (3.99 mg/g) in the methanolic leaf extract [72]. Another investigation using an ultrasonic-assisted methanolic extraction of the powdered plant yielded 3.96 mg/g of ursolic acid per dry weight of plant material [73]. Furthermore, the hot water extract demonstrated the presence of gallic (0.19 mg/mg), chlorogenic (0.17 mg/mg), caffeic (0.66 mg/mg), coumaric (1.7 mg/mg), rutin (0.20 mg/mg), and rosmarinic (2.41 mg/mg) acids [45]. Interestingly, extracts prepared from air-dried *P. amboinicus* showed a higher total content of phenolic compounds and superior antioxidant activity compared to those made from fresh plants. Caffeic acid content was generally higher in the fresh material. Conversely, rosmarinic acid was the predominant phenolic acid in dried material [74].

There have been several studies that have showcased the varying compositions of secondary metabolites present in the same species yet grown in different climates. One such example investigated *P. amboinicus* that had been cultivated in different locations, including Cianjur and Bogor in Indonesia and Poznan in Poland. The Indonesian plants were grown in a tropical climate, while the Polish plants grew in a moderate climate. An analysis of polyphenolic and diterpene content using LC-HRMS, after extraction with methanol, found that the leaves of Polish plants contained the highest equivalents (112.95 ± 0.8) mg/g of gallic acid (polyphenol) compared to Indonesian plants (23.61 ± 0.2 and 16.79 ± 1.5 mg/g). As for the diterpene content, Indonesian plants contained higher equivalents of carnosic acid (23.64 ± 0.2 and 15.59 ± 0.2 mg/g) compared to less than 1.0 mg/g in the Polish sample. The major diterpenes found in Indonesian plants were acetoxydihydroxyroyleanone and dihydroxyroyleanone, whereas in Poland, rosmanol and rosmadial were the most abundant diterpenes. Evidently, the suboptimal climate in Poland likely upregulated phenolic biosynthesis at the expense of diterpenoid pathways. Longer photoperiods and lower temperatures favored phenolic accumulation in Poland, demonstrating that climatic and geographical conditions significantly influence the phytochemical profile of *P. amboinicus* [75].

#### 2.1.2. Stems

Research groups with samples from Taiwan, India, and Poland have studied the composition of MeOH extract from the stems of *P. amboinicus* [72,76]. After solvent partitioning and chromatographic separation, the sample from Taiwan afforded four flavonoids, four benzenoids, and three steroids, as well as one new compound that was identified as 5,8-dihydroxy-7,2′,3′,5′-tetramethoxyflavone. The extracted flavonoids were 5-hydroxy-7,2′,3′,5′-tetramethoxy-flavone, 5-hydroxy-7,3′,4′-trimethoxyflavone, 5,3′-dihydroxy-7,4′-dimethoxyflavone, and 5,4′-dihydroxy-7,3′-dimethoxyflavone. The benzenoids were vanillic acid, p-hydroxybenzoic acid, methylparaben, and caffeic acid. Finally, the three steroids were β-sitosterol, β-sitostenone, and stigmastenone [76]. In the MeOH extract from the plant from Mysore, India, flavonoids, such as rutin and quercetin, were also detected, which represented 26.6 mg rutin equivalents per gram of extract (REs/g extract). Hydroxycinnamic acids, namely, caffeic acid, *p*-coumaric acid, and gallic acid, were also documented in the stem extract taken from India. Bhatt et al. (2013) also reported a total phenolic content of 49.91 mg gallic acid equivalents per gram of extract (GAEs/g extract), and a content of proanthocyanidins of 0.7 mg tannic acid equivalents per gram of extract (TAEs/g extract) [72]. Another group also studied the composition of the MeOH extract from the stems of plants cultivated in Poland, a colder climate than the one the plant is usually grown in [75]. The stems were rich in rosmanol (0.19 mg/g), acid-digestible fiber (ADF) and ash neutral detergent fiber (aNDF). Other nutritional components such as dry matter, organic matter and crude ash were also reported. The benzenoids vanillic acid and caffeic acid, as well as syringic acid, were also detected. Moreover, verbascoside, rosmarinic acid, tubuloside B, melitric acid, salvianolic acid, luteolin, epirosmanol carnosol, and dihydroxyroyleanone were recorded. Total phenols were reported as 16.55 ± 0.9 mg GAEs/g extract, phenolic acids as 12.78 ± 1.1 mg rosmarinic acid equivalents (RAEs)/g extract, flavonoids as 14.21 ± 1.1 mg isoquercitrin equivalents (IEs)/g extract, and diterpenes as 0.41 ± 0.02 mg carnosic acid equivalents (CAEs)/g extract [75].

In an additional study, the quantification and identification of the major active constituents in the air-dried stems of *P. amboinicus* from Karnataka, India, was performed. The study revealed that total phenolics represented 14.30 ± 0.49% *w*/*w*, flavonoids 3.59 ± 0.41% *w*/*w*, alkaloids 1.28 ± 0.50% *w*/*w*, and saponins 0.84 ± 0.15% *w*/*w*. Moreover, total ash represented 11.43 ± 0.02% of the stem part, water soluble ash value 5.85 ± 0.22%, acid insoluble ash value 0.95 ± 0.18%, and loss on drying 9.30 ± 0.10%. All these values were higher for leaves than for the stems, and the stem’s values were higher than those of the roots [36].

Furthermore, a study by Stasińska-Jakubas et al. (2023) compared chitosan suspension and chitosan lactate solution for stimulating phenolic compounds in *P. amboinicus* [63]. Chitosan, a natural polysaccharide biopolymer, is increasingly used as a biotic elicitor for optimizing plant production. The research group concluded that chitosan affected polyphenol content in leaves and stems, and that anthocyanins in stems decreased with both forms. Chitosan did not affect total phenolic compounds and flavonols in stems, but chitosan lactate reduced free-radical-scavenging activity. Moreover, Chitosan lactate increased stem dry weight by 15%. Their results suggest that chitosan treatment might not be the most effective at increasing desired phytochemicals in the stems.

#### 2.1.3. Essential Oil (EO)

Essential oils (EOs) are a class of natural aromatic oils extracted from different plant parts, including leaves, stems, bark, buds, fruits, and flowers. They are composed of a variety of volatile compounds that belong to different chemical classes, including alcohols, aldehydes, amides, amines, ketones, oxides, phenols, and terpenes [77,78,79]. Due to their known wide range biological activities with diverse mechanisms of action, including hepatoprotective, antioxidant, antispasmodic, anti-ulcerogenic, anticancer, antimicrobial, anti-inflammatory, and antidepressant effects. With mechanisms such as enhancing the permeability of microbial cells through the degradation of the cell wall, disruption of the cytoplasmic membrane, damage to membrane proteins, and coagulation of cellular proteins, EOs have established themselves as significant therapeutic agents over the years. Furthermore, plant-derived products, and particularly EOs, have played crucial roles in modern drug development, especially as antimicrobial and antitumor agents. Some authors suggest that the EO of *P. amboinicus* (EO-Pa) and its components, specifically, could be utilized to develop new and more effective antimicrobial and anticancer agents [74,78,79].

The chemical composition of EOs is determined by genetic factors. However, the secondary metabolites produced and the yield are affected by a plethora of biotic and abiotic factors, including plant–microorganism, plant–insect, and plant–plant interactions, geographical origin, plant part, seasonal variation in the chemical isomer structures, age and development stage of the plants, harvest time and season, luminosity, temperature, rainfall, extraction conditions, extraction methods, drying, storage, and analysis methods [17,79,80,81,82]. For example, research by Ślusarczyk et al. (2021) aimed to compare the phytochemical composition of *P. amboinicus* plants from Indonesia and Poland [75]. Polish plants, adapting to a suboptimal climate with a longer photoperiod and lower temperatures, showed increased phenolic biosynthesis compared to their Indonesian counterpart, and they also showed decreased biosynthesis of diterpenoids and terpenoids. The group also determined that temperature impacts the metabolic profile, with higher temperatures boosting both terpenoids and phenolics. Additionally, according to a study by Sabra et al. (2018), optimal oil production by *P. amboinicus* is achieved with long days and high light intensities [83]. However, excessive light and extreme temperatures will negatively impact the growth of the plants. The research group also determined that the length of the irrigation intervals that are ideal for increased plant production depend on the time of the year [83].

Furthermore, extraction conditions also play an important role for the quality of the oil. Rapid distillation results in a product rich in volatile constituents with superior organoleptic and chemical characteristics. In contrast, prolonged extraction increases costs and may lead to the presence of less desirable compounds [80]. A study by Lopes et al. (2017) [80] demonstrated that the efficiency of extraction by steam distillation of the leaves of *P. amboinicus* increased after the 3 h mark, and after this point, the extraction remained steady [80]. Moreover, the chemical constituents of EO-Pa vary depending on the geographical origin of the samples. Recently, Monzote et al. (2020) reported that more than 70 components of the EO-Pa had already been described in different locations [74].

Interestingly, Monzote et al. (2020) [74] also reported three distinct chemotypes for the EO-Pa, which are the carvacrol-/thymol-rich and carvacrol-/thymol-poor chemotypes. The carvacrol-rich chemotype is predominant, with an average of 69.4% carvacrol, ranging from 32.3% to 98.0%. The thymol-rich chemotype had an average of 67.3% thymol, ranging from 35.0% to 94.3%, and low concentrations of carvacrol. Finally, the carvacrol-/thymol-poor chemotype exhibits low concentrations of both carvacrol and thymol, both under 11%. This chemotype distribution appears random and independent of geographical location, as all three chemotypes are present in samples from different countries in different continents [74]. These results indicate that many factors do indeed contribute to EO quality and quantity and that, possibly, geographical location is not the most predominant one.

In summary, the production and composition of EO-Pa are influenced by various environmental, genetic, and geographical factors. Understanding these influences is essential for optimizing oil yield and quality, making extraction method, irrigation intervals, and harvest times crucial parameters for achieving desired results. The variability in chemotype distributions across different regions further highlights the importance of considering local conditions in essential oil production.

The following section has been divided into two subsections, to explore how different plant parts produce EOs with different compositions. Due to the large number of reports on extracts exclusively from the leaves of EO-Pa, we felt it necessary to group the stem, aerial parts, and whole plant into one section and the leaves into a separate section.

##### Stem, Aerial, and Whole Plant EO

To afford EO-Pa, both steam distillation and hydrodistillation are employed; however, most reports indicate hydrodistillation for 2 h–5 h as the extraction method. Current data do not suggest that one method produces a higher oil yield, and there were no mentions regarding the color or appearance of the oil in the reviewed literature for the EO-Pa of the stems, aerial parts, and whole plant [37,46,74,77,81,83,84,85,86].

The stem EO-Pa showed a carvacrol-rich chemotype (Table 1). Four main components were identified in this oil from Brazil, representing 100% of the oil contents. The identified metabolites were carvacrol (76.33%), Caryophyllene (9.33%), Terpinene (8.99%), and ρ-Cymene (5.35%). These results are interesting because most EO-Pas present a higher diversity in the oil content. The group also reported a density for the EO, which was 0.9365 g/mL [46].

Regarding the chemotype, most samples (Table 1) presented the carvacrol-rich chemotype, with carvacrol percentages in the carvacrol-rich chemotype between 17.9% and 88.17%. Interestingly, all nine samples studied in Table 1 had carvacrol (0.45–88.17%) [37,46,74,77,81,83,84,85,86].

The thymol-rich chemotype was less predominant and was only observed in three samples: two from Egypt [83], with percentages much lower than those of the carvacrol in the carvacrol-rich chemotype, and one from India [86]. For the plants from Cairo, Egypt, the thymol documented represented 32.4% and 34.89% of the oil content from the plants harvested in February (4-day irrigation interval) and August (16-day irrigation interval), respectively [83]. The thymol present in the EO-Pa from India corresponded to 83.39% of the oil. Two other EO-Pa samples had thymol, but in lower quantities (0.3% and 0.21%).

The carvacrol-/thymol-poor chemotype was observed in only one sample, from Cairo, Egypt [84]. The sample only had 5.96% carvacrol, and no thymol was reported. Its main component was γ-Terpinene, constituting 8.64%. Indeed, γ-Terpinene is a common component of the EO-Pa of stems, aerial parts, and whole plants, being reported seven out of nine times as a component in the reviewed literature used for Table 1, with values ranging from 0.45% to 18.86%. In general, higher values were observed when whole plants were used for extraction of the EO-Pa instead of only aerial parts [37,46,74,77,81,83,84,85].

ρ-Cymene was reported in almost all samples [37,46,74,77,81,83,84,85], except for the sample from India [86], with percentages between 0.72% and 13.65%. EO-Pa from both aerial and whole plants had high percentages of ρ-Cymene. Most samples also contain β-Caryophyllene (0.71–14.98%), Caryophyllene oxide 0.37–5.85%), α-Terpinene (0.22–4.1%), α-Humulene (0.35–3.24%), Myrcene (0.53–2.45%), Oct-1-en-3-ol (0.6–3.25%), and α-Thujene (0.41–0.8%). Several samples also contained Terpinen-4-ol (0.93–2.93%), Terpinolene (0.63–1%), α-Cadinol (0.4–5.67%), and β-Pinene (0.68–1.1%) [37,46,74,77,81,83,84,85,86].

In general, more distinct compounds were identified in the EO-Pa of whole plants than the EO-Pa of only aerial parts or stems. Stem EO-Pa had the lowest number of reported compounds, with only four natural products identified [46]. In the case of the EO-Pa of the whole plant, between 16 and 33 compounds were identified (average = 22.5) [77,83,84], and for the EO-Pa of aerial parts, between 6 and 14 compounds were detected (average = 11.5) [37,74,81,86], with percentages above 0.2%. In total, over fifty different compounds have been identified for the EO of the stems, aerial parts, and whole plants of *P. amboinicus*. In many plants, different types of bergamotenes, cadinols, caryophyllenes, humulenes, pinenes, and terpinenes have also been documented.

Moreover, research by Roja et al. (2006) demonstrated that tissue cultures of *P. amboinicus* can be a significant source of thymol and cis-caryophyllene [85]. In the study, EOs were extracted from parent plant tissue, tissue-cultured plants, and roots from cultured plants and then analyzed by GC-MS. GC-MS analysis revealed 21 constituents in parent plants and root cultures and 15 in tissue culture plants, which were identical to those in the parent plants. Key compounds identified were thymol and cis-caryophyllene. The thymol content was 0.009% in the parent plant, 0.012% in tissue-cultured plants, and 0.29% in root cultures. Root cultures showed a threefold increase in thymol content over 25 days. This suggests tissue cultures, especially root cultures, as a viable source of thymol and cis-caryophyllene, comparable to *Thymus vulgaris*. However, it must be noted that there is a loss of diversity of compounds when using this method of production.

##### Leaf EO

There are two predominant methods of leaf essential oil preparation in the reviewed literature: extraction by steam distillation and by hydrodistillation. Hydrodistillation using a Clevenger apparatus is the most common method. In the literature, the extraction times ranged between 2 h and 6 h, and the obtained oil was described as pale-yellow or light-yellow [17,21,31,32,33,48,58,78,79,80,82,87,88,89,90,91,92]. However, one group reported a colorless oil [87]. Jena et al. (2023) also reported the refractive index and density of the EO, obtained by hydrodistillation, to be 1.48 and 0.105 g/mL [88]. This density is similar to the one reported for the EO-Pa of the stems [46,79]. Lopes et al. (2017) reported a refractive index of 0.9167 ± 0.04 N_D_ and a density of 1.5 g/mL for the EO-Pa obtained by steam distillation [80]. Differences may arise from the different methods of extraction.

The chemotype that is most often reported is the carvacrol-rich one. Across twelve studies, with fourteen samples in total (Table 2), carvacrol was present in nine samples and was the predominant component in eight of them. The percentage of carvacrol ranged from 1.2 to 88.61% and was detected as the predominant phytochemical in samples from Malaysia, Cambodia, India, and Brazil [31,48,82,87,88,89,90,92].

The thymol-rich chemotype was also reported in three samples. The samples from Brazil had 45.64 and 64.3% thymol, and the sample from India had 94.3% thymol. Moreover, out of the fourteen plants, eight had thymol as one of the components of the EO of the leaves, with the percentages ranging between 0.3 and 94.3% [17,31,48,78,87,88,89,90]. Interestingly, the hydroxylation of ρ-Cymene, another common natural product of *P. amboinicus*, leads to the formation of thymol [17]. It is also noteworthy that it is known that thymol is heat-sensitive, and a study conducted on the extraction kinetics of thymol from *P. amboinicus* by ultrasonic-assisted extraction concluded that the optimal extraction temperature was 55 °C, with lower amounts of the secondary metabolite detected as the temperature increased [91]. Many studies conducted on the EO-Pa use hydrodistillation as the extraction method, which commonly uses temperatures of 100 °C. This can influence the composition of the EO-Pa, greatly affecting the percentage of thymol and other heat-sensitive secondary metabolites.

In general, the percentage of thymol in the thymol-rich chemotypes was lower than the percentage of carvacrol in the carvacrol-rich chemotype. It is also noteworthy that, in one sample, the percentage of both thymol (20.17%) and carvacrol (20.25%) was almost identical.

Finally, the carvacrol-/thymol-poor chemotype was also reported by two different groups, with samples with neither carvacrol nor thymol present. In the sample from Vishnu et al. (2021), the main component was Caryophyllene (10.78%) [79], and in all three samples from Gonçalves et al. (2012), terpinen-4-ol was the main component, with percentages of 90.55, 95.39, and 98.03% [21]. Even for the three EOs produced by [21] from *P. amboinicus*, which were obtained in three different periods, there are notable differences between all samples. While the percentage of Terpinen-4-ol is similar across all samples, some other compounds such β-Caryophyllene and α-Bergamotene were detected in only one of the three samples, and even when one compound was detected in more than one sample, its percentages varied significantly between samples. For example, Caryophyllene oxide was detected in two samples with percentages of 1.36 and 0.2% [17,21,31,48,58,78,79,80,82,87,88,89,90,92]. The results, summarized in Table 2, clearly show an immense level of variation of the phytochemicals detected in each plant, and there is no clear relationship between the extraction method, time, and region in which the plant was grown with the type and number of phytochemicals present in the EO-Pa of the leaves.

More than 100 unique compounds have been extracted from the EO of the leaves of *P. amboinicus* (Table 2). Interestingly, the vast majority of these natural products are reported only once in the reviewed literature, meaning that they were unique to that sample. Conversely, no compound is reported in all samples. Moreover, even though *P. ambonicus is* well known for being rich in carvacrol and thymol, these compounds are not always present, and they are not the ones that are most often reported. Indeed, the compound that is most often seen across all fourteen EO-Pa samples is caryophyllene oxide, which was reported 10 times with a concentration ranging from 0.2 to 10.78%. Other caryophyllenes, such as β-caryophyllene and (*Z*)-caryophyllenne, are also reported across many samples. In fact, β-caryophyllene was reported in five different samples from Asia and America, with percentages between 1.9 and 8.9%. Terpinen-4-ol is also a common compound in the EO of the leaves of *P. amboinicus*, with eight reports that ranged between 0.2 and 98.93%. ρ-cymene and γ-terpinene were also reported eight times, with percentages between 0.3 and 28.2% and between 0.56 and 14.77%, respectively. Another natural product often extracted from *P. amboinicus* is β-myrcene, reported seven times (0.33–12.59%). Additionally, (*E*)-β-farnesene (0.2–1.55%), α-humulene (0.33–9.67%), and α-pinene (0.2–0.38%) were also documented in several samples.

Several types of aromadrendes, bergamotenes, carenes, other types of caryophyllenes, farneses, humulenes, pinenes, selinenes, and terpinenes were identified across all samples. In fact, a type of caryophyllene was reported in almost all samples, except for the one by Singh et al. (2002) [48]. Most notably, one or several types of terpenes were reported for all samples. It is also important to consider that, in several of these extracts, a small percentage of the EO composition could not be identified, which could lead to under-reporting of some species. It is also possible that the number of compounds present in each plant varies greatly. For example, Lopes et al. (2017) obtained 19 components in the EO-Pa, which accounted for 99.99% of the oil composition [80]. On the other hand, Kweka et al. (2012) documented 26 compounds that represented 99.93% of the oil composition [89]. Moreover, 14 compounds accounted for 97.35% of the oil composition according to Santos et al. (2015) [87], and Jena et al. (2023) [88] identified an astonishing 57 compounds. It is also important to consider that the extraction method greatly affects the quality and quantity of EO obtained, and it has been documented that a minimum of 3 h of steam distillation are necessary for better yields [80]; however, several studies only performed hydrodistillation for 2 h [21,32,78]. Nonetheless, it is clear that *P. amboinicus* can produce a remarkable variety of phytochemicals [57,86,87,91].

##### Comparison of EOs

The most common component reported in the EO-Pa of both leaves and stems/aerial parts/whole plants is clearly carvacrol, and although many reports highlight the rich content of thymol of some of the specimens of these plants, the second most common compound is caryophyllene oxide, followed by ρ-cymene, then γ-terpinene, and finally thymol. This indicates that *P. amboinicus* might be a more reliable source of other components, albeit present in lower quantities. Other common compounds are terpinen-4-ol, β-myrcene, β-caryophyllene, α-humulene, α-terpinene, and α-thujene. Interestingly, oct-1-en-3-ol was reported more often for the EO-Pa of aerial parts and whole plants than for the EO-Pa of leaves. Other common compounds of the EOs-Pa include α-terpinolene and α-Pinene; however, these were reported less often than those previously described. Notably, ρ-cymene was present in almost all samples of the EO-Pa of stems/aerial parts/whole plants, while being present in only half of the EOs-Pa of leaves, which might indicate that the leaves are not the main source for this compound. Two types of cadinol were reported in the EO-Pa of the stem/aerial parts/whole plants, while there were no reports of the compound in the leaf EO-Pa [17,21,31,32,33,37,46,48,58,74,77,78,79,80,81,82,83,84,86,87,88,89,90,91,92].

### 2.2. Biological Activities of P. amboinicus

**Figure 1:**
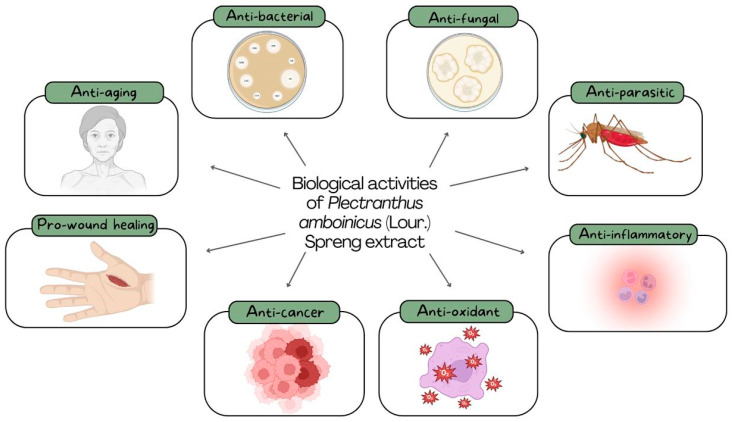
Scheme of the biological activities of *Plectranthus amboinicus*.

Plectranthus amboinicus has garnered significant scientific interest due to its broad spectrum of pharmacological activities. These include antimicrobial (antibacterial, antifungal, and antiparasitic), anti-inflammatory, antioxidant, anti-aging, and wound healing effects, as well as anticancer potential. Figure 1 provides a visual overview of the principal biological properties attributed to *P. amboinicus* extracts, as reported in recent studies

#### 2.2.1. Antibacterial Activity

*P. amboinicus* extracts and essential oils have demonstrated considerable antibacterial activity against a wide range of bacterial strains, including Gram-positive and Gram-negative pathogens. Some research has studied this activity by utilizing various extraction methods and solvent systems. Previously, Aparna and Gayathri (2018) focused on developing a natural medicine using culinary herbs and aloe vera gel to address bacterial skin infections caused by Gram-positive cocci such as *Staphylococcus aureus*, *Staphylococcus epidermidis*, and *Streptococcus pyogenes* [53]. They developed methanolic, acetone, and aqueous extracts of *P. amboinicus*, *Mentha piperita*, and *Ocimum basilicum* and investigated their antibacterial activity using the agar well diffusion method. The methanolic extract of the herbal combination, notably *P. amboinicus*, displayed a substantial inhibitory effect against the bacterial strains, with *S. epidermidis* being the most sensitive. Incorporating aloe vera gel into the herbal mixture demonstrated promising synergistic effects, leading to the development of an effective natural medicine formulation against bacterial skin diseases.

#### 2.2.2. Antifungal Activity

Recent scientific investigations have shed light on the antifungal activities of *P. amboinicus*, showing its potential as a natural source for the production of antifungal medicines [46,48,58,93], particularly against dermatophytes causing skin infections [48]. Exploring the antidermatophytic potential of medicinal plants, Chellappandian et al. (2018) studied 18 species, including *P. amboinicus* [94].

The ethanolic extract of *P. amboinicus* demonstrated notable antibacterial activity, particularly against several Gram-positive and Gram-negative pathogens. Phytochemical screening identified bioactive compounds including alkaloids, terpenoids, tannins, and phenolics, as well as specific constituents like luteolin and verbascoside. The extract exhibited inhibitory effects against eight out of seventeen tested bacterial strains, with *S. aureus* strain PB57 (MRSA) showing the highest susceptibility (inhibition zone: 11 mm). The MIC and MBC values were 7.81 mg/mL and 15.63 mg/mL, respectively. However, cytotoxicity assays using human fibroblasts indicated that concentrations close to or above the MBC significantly reduced cell viability, with an IC_50_ value of 541.2 µg/mL. These results suggest that while the extract has promising antimicrobial potential, its cytotoxicity at higher concentrations may limit direct therapeutic use, warranting further fractionation or structural modification to enhance safety profiles [95].

The essential oil of *P. amboinicus*, primarily composed of thymol (approximately 70.7%), demonstrates strong antibacterial activity against *Klebsiella pneumoniae*, with a minimum inhibitory concentration (MIC) of 400 μg/mL. In vitro assays confirmed its bactericidal nature, showing complete growth inhibition and metabolic inactivity at MIC levels. Proteomic analysis revealed that the oil disrupts key bacterial systems, including antioxidant defense and efflux pumps, leading to oxidative stress and membrane destabilization. This was evidenced by the downregulation of resistance-related proteins such as ompA (−24.3-fold), acrB (−21.7-fold), and oxa2 (−8.4-fold), alongside upregulation of stress-response proteins like nuoF and trxA. These molecular effects were supported by microscopy, FTIR, and biochemical assays, which showed structural membrane damage, leakage of nucleic acids and proteins, and elevated reactive oxygen species levels. The oil also significantly disrupted mature biofilms and reduced the expression of biofilm-associated genes. Cytotoxicity assays indicated it was non-toxic to human lung epithelial A549 cells up to 400 μg/mL. In vivo, the oil was well tolerated in zebrafish up to 12.5 μg/mL, where it enhanced survival, reduced bacterial load, and minimized tissue damage following infection. These results collectively demonstrate the oil’s multi-targeted antimicrobial action and its potential as a complementary therapy against drug-resistant *K. pneumoniae* infections [96].

The essential oil of *P. amboinicus* showed broad-spectrum antimicrobial and antibiofilm activity, targeting major human pathogens including drug-resistant strains. Compositional analysis identified thymol as the dominant constituent, contributing to its strong antimicrobial properties. The extract was effective in disrupting mature biofilms and exhibited significant bactericidal effects, including in resistant strains such as *Klebsiella pneumoniae*. In vitro tests demonstrated dose-dependent inhibition of bacterial growth, while transcriptomic analyses indicated downregulation of genes associated with virulence, quorum sensing, and membrane integrity. Notably, the oil was also evaluated for its anti-inflammatory and antioxidant activities, reinforcing its therapeutic potential. These findings support the use of *P. amboinicus* as a source of natural compounds with multiple biological activities relevant to combating antibiotic resistance and microbial persistence mechanisms [97].

A recent study by Saravanan et al. (2025) [67] explores the green synthesis of copper oxide nanoparticles (CuONPs) using *P amboinicus* leaf extract and evaluates their antibacterial properties against Gram-negative bacteria. Characterization techniques, including UV-Vis spectroscopy, FTIR, XRD, SEM, and EDX, confirmed the formation of cubical CuONPs with a characteristic UV-Vis absorption peak at 325 nm and the presence of copper and oxygen elements. These nanoparticles exhibited significant bactericidal activity against *E. coli* and *Shigella flexneri*. In silico molecular docking studies further revealed that bioactive compounds from the leaf extract, such as alpha-calacorene and calamenene, have a strong binding affinity to the BamA protein, a crucial component in the outer membrane protein assembly of Gram-negative bacteria, suggesting a potential mechanism for the observed antibacterial effects. The findings indicate that biogenically synthesized CuONPs, along with specific phytochemicals from *P. amboinicus*, hold promise as effective antibacterial agents against Gram-negative pathogens [67].

#### 2.2.3. Mosquito Repellent and Anti-Parasitic Effects

A study by Lalthazuali and Mathew (2017) highlighted the extraordinary mosquito repellent and anti-parasitic effects of essential oils extracted from *P. amboinicus* and other aromatic plants [98]. A 20% ethanolic *P. amboinicus* oil solution displayed repellency against Aedes aegypti mosquitoes, vectors of dengue, chikungunya, and yellow fever, comparable to DEET for up to 6 h. A 5% synergistic mix combining *P. amboinicus*, *Ocimum sanctum*, *Mentha piperita*, and *Eucalyptus globulus* oils likewise showed substantial 6 h repellency, reflecting DEET’s performance. Furthermore, *P. amboinicus* displayed strong anti-parasitic efficacy against *Plasmodium falciparum*, *Giardia lamblia*, and *Entamoeba histolytica*, attributed to bioactive substances including carvacrol and thymol, highlighting its potential for eco-friendly vector control and anti-parasitic therapy.

Elucidating the phytotherapeutic potential of *P. amboinicus*, El-Gohary et al. (2019) evaluated the effects of selenium and humic acid treatments on its mass production, essential oil content, and antibacterial activity [84]. These treatments greatly enhanced plant biomass and essential oil yield, with carvacrol (5.96–15.45%) being the predominant ingredient, followed by γ-terpinene and limonene. Notably, the treatments improved the antioxidant activity of *P. amboinicus*, highlighting its potential as a natural antioxidant source. While precise antibacterial data were not provided, the study underlines the antimicrobial efficacy of *P. amboinicus*, related to its rich phytochemical makeup, notably essential oil constituents like carvacrol, γ-terpinene, and limonene. These findings underline the potential of *P. amboinicus* as a natural source of antibacterial and antioxidant compounds for treating skin infections and infectious disorders.

#### 2.2.4. Anti-Inflammatory Activities

*P. amboinicus* has been the focus of intensive studies due to its rich metabolites with potential anti-inflammatory activity [69]. Traditionally used as an anti-inflammatory agent [49], the aqueous extract of *P. amboinicus* has been reported to exert significant anti-inflammatory effects [63]. These effects include the blockage of NF-*κ* B activation, which would consequently reduce the production of pro-inflammatory cytokines [69].

A study highlighted the significant anti-inflammatory and protective benefits of the aqueous leaf extract of *P. amboinicus* against lipopolysaccharide (LPS)-induced endotoxemia and inflammation in a rat [66]. The extract was notably effective in mitigating the harmful effects of LPS, including the inhibition of pro-inflammatory cytokines TNF-α and IL-8 and correction of hematological abnormalities such as anemia and leukopenia. Pre-treatment with the *P. amboinicus* extract demonstrated its preventive significance, alleviating the detrimental impacts of LPS exposure. The extract also displayed a strong protective effect on the kidney and liver, as evidenced by decreased renal indicators and reduced hepatic enzyme activity. This study suggests that *P. amboinicus* extract holds promise as a natural and non-toxic therapeutic option for combating endotoxemia-induced inflammation and toxicity [55]. These findings also support the traditional use of *P. amboinicus* in folk medicine, underscoring its potential as a valuable addition to modern therapeutic practices.

#### 2.2.5. Antioxidant Activities

Research has demonstrated that the significant antioxidant properties of *P. amboinicus* plant extracts are largely due to their rich polyphenolic content, which effectively neutralizes free radicals and mitigates oxidative stress. This can help prevent skin conditions like hyperpigmentation, premature aging, and acne management [99].

The stem of *P. amboinicus* exhibits high antioxidant potential, as evidenced by selected in vitro assays. The methanolic extract of Indian borage stems demonstrated a DPPH free-radical-scavenging activity of 96% at 200 ppm, surpassing the 72.7% activity reported for the aqueous extract of the leaves at 300 ppm by Kumaran and Karunakaran (2006) [100]. Previous research has established a direct correlation between antioxidant activity and reducing power ability of certain plant extracts [72].

Carvacrol and thymol, phenolic monoterpenoids found in various essential oils, have numerous practical applications in food and medicine due to their wide range of biological activities, including antioxidant properties [101,102,103,104,105]. Essential oils with antioxidant activity, therefore, could be beneficial in acne management. Recent studies have shown that *P. amboinicus* essential oil exhibit strong antioxidant activity in various in vitro assays [99].

In addition to scavenging free radicals, *Plectranthus amboinicus* exerts its antioxidant effects by upregulating key antioxidant enzymes and regulators. Specifically, the extract activates the Nrf2 (nuclear factor erythroid 2–related factor 2) pathway, which in turn enhances the transcription of downstream cytoprotective gene catalase [103]. This mechanistic pathway not only supports its role in neutralizing reactive oxygen species but also underpins its protective effects against oxidative stress-induced skin aging.

#### 2.2.6. Skin Enzymes

The degradation of the extracellular matrix (ECM) is primarily driven by the increased activity of proteolytic enzymes such as collagenase and elastase. Natural plant compounds that inhibit these enzymatic activities present a promising approach to preventing skin aging. These compounds are becoming increasingly important as ingredients in cosmetics and medications designed to combat skin aging [99].

*Plectranthus amboinicus* extract has demonstrated significant inhibitory effects on tyrosinase and collagenase enzymes, which are crucial for protecting the skin against hyperpigmentation and premature aging. Ito J. et al. (2018) conducted a comprehensive study on the inhibitory effects of extracts from 25 plants obtained in Sri Lanka, including *P. amboinicus* [106]. Their findings highlighted that *P. amboinicus* extract effectively inhibits both tyrosinase and collagenase enzymes. Similarly, Jugreet et al. (2022) reported notable anti-collagenase activity, with an IC_50_ of 0.33 ± 0.02 µg/mL [99]. Additionally, Ito J. et al. (2018) studied the inhibitory effects of *P. amboinicus* 70% ethanolic extract, which displayed tyrosinase and collagenase inhibition percentages of up to 50%. These results further support its potential as a valuable ingredient in skincare formulations [106]. The essential oil of *P. amboinicus* exhibited high collagenase inhibition, quantified with an IC_50_ of 0.33 ± 0.02 mg/mL [99].

#### 2.2.7. Insecticide

The essential oil of *P. amboinicus* has shown insecticidal activity against various insect species, including the stable fly (*Stomoxys calcitrans*) and the horse fly (*Tabanus megalops)* [107]. Previous studies have demonstrated that both contact and fumigant toxicity tests exhibited the lethal effect of *P. amboinicus* essential oil against adult *S. calcitrans* and *T. megalops* in a dose-dependent manner [107,108]. The essential oil at low concentrations of 9.3, 18.7, and 37.4 µg/µL showed similar mortality to the negative control (acetone) in the contact toxicity test against *S. calcitrans*, whereas the essential oil at high doses of 46.7 and 93.4 µg/µL showed similar mortality to the positive control (cypermethrin 1%) [107,108]. However, the insecticidal efficacy was rather low during the first hours of observation after the exposure and was approximately 90% at 24 h. The insecticidal efficacy of the essential oil is influenced by the dose and time of exposure. In terms of toxicity values obtained in the present study, the LD_50_ and LC_50_ of the *P. amboinicus* essential oil against *S. calcitrans* at 24 h after treatment for the contact and fumigant toxicity tests were 12.05 µg/fly or 2.41% (*w*/*v*) and 1.34 mg/L air or 1.34 µg/cm^3^ air, respectively. The efficacy of other essential oils against this species has been documented; for example, the essential oil from catnip (*Nepeta cataria*) showed an LD_50_ and LC_50_ of 16.4 µg/fly and 10.7 mg/cm^3^ against *S. calcitrans*, respectively [109].

The essential oil of *P. ambonicus* showed larvicidal activity against *Anopheles stephensi* reared in the laboratory with LC_50_ values of 33.54 (after 12 h) and 28.37 ppm (after 24 h). The larval mortality ability of the essential oil may be accredited by the presence of carvacrol in the essential oil [33]. The results of the present study are also similar to that of earlier investigations on the larvicidal activity of essential oils.

#### 2.2.8. Cytotoxicity

The extract obtained from *P. amboinicus* demonstrated cytotoxic activity against the HeLa cell line [110]. A study employing HPLC-based metabolomics was conducted to identify cytotoxic compounds from *P. amboinicus* against human breast cancer MCF-7 cells [59]. The decrease in viable cell counts and the simultaneous rise in non-viable cancer cell count, shifting towards normal levels in the tumor host, indicate an apparent antitumor effect against EAC (Ehrlich Ascites Carcinoma) and DLA (Dalton’s Lymphoma Ascites) cells in mice [111]. The anticancer study’s results indicated that the methanol extract derived from *P. amboinicus* leaves exhibited notable dose-dependent cytotoxicity, demonstrating remarkable anticancer activity against the tested cell lines, particularly DLA.

The methanol extracts of *P. amboinicus* demonstrated cytotoxicity percentages of 4.2, 12.6, and 30.4% in DLA cell lines at concentrations of 50, 100, and 200 µg/mL, respectively. The in vitro anticancer study on DLA cell lines revealed high activity with increasing concentrations of the extract. Notably, no cell death was observed at lower concentrations (10 µg, 20 µg). The in vitro anticancer study on DLA cell lines revealed high activity with increasing concentrations of the extract. Notably, no cell death was observed at lower concentrations (10 µg, 20 µg). As the concentration of the extract increased, a significant decrease in viable cells was observed. The IC_50_ value of *P. amboinicus* against oral cancer cells was determined to be 53 µg/mL [112].

The ethanolic extract of *P. amboinicus* leaves contains flavonoid compounds that demonstrate exceptionally potent anticancer and antioxidant activities. Consequently, the ethanolic extract from these leaves exhibits robust antioxidant properties [113]. When administered to C57BL/6 mice via injection, the essential oil of *P. amboinicus* demonstrated a robust chemotherapeutic effect on the B16F-10 melanoma cell line [114]. Calculated IC_50_ values for cytotoxic activity against breast (MCF-7) and colorectal (HT-29) cell lines were 53 ± 0.01 mg/mL and 87 ± 0.01 mg/mL, respectively [115]. The current study primarily focused on DLA cell lines, without examining other carcinomas. Therefore, a more detailed investigation involving various types of cancer cells is warranted using the same plant extract. The extracts were prepared in methanol; however, exploring additional combinations in different solvents is essential to comprehending the broader potential effects of the plant on various carcinomas. The results obtained in the present investigation are highly significant, particularly given the limited exploration of specific cancer cells against *Plectranthus* leaf extracts. This information could prove invaluable for future studies, allowing for comparisons with other types of cancer cells. Notably, DLA and EAC, being important cancer cell types, have garnered increased attention and importance in ongoing research. The comprehensive exploration of this plant is imperative to uncovering a multitude of remarkable compounds within it. One such example is the isolation of a novel abietane diterpene, 16-acetoxy-7-α,12-dihydroxy-8,12-abietadiene-11,14-dione, which has been successfully identified in the acetone extract of the root of *Plectranthus hereroensis*. This discovery underscores the potential for further in-depth investigations to reveal additional noteworthy compounds within the genus [116]. Previous studies that reported no signs of toxicity and mortality after treatment with *P. amboinicus* extract at a dose of 2000 mg/kg (acute) or 200 and 400 mg/kg (subacute) [117]. Another study conducted on *P. amboinicus* extract by Borg Karlsson et al. (2014) [118] showed no mortality at a 5000 mg/kg dose (acute) and at doses of 2500, 1250, and 625 mg/kg (subacute) of the aqueous extract for 28 days, although treatment-related toxicological abnormalities, such as necrosis and hemorrhages, increased with the dose. The toxicity reported could be attributed to overdosing [118]. These results highlight the importance of adhering to the dose limit prescribed by herbalists on the basis of the patient’s health and physique.

The cytotoxic effects of *Plectranthus amboinicus* on cancer cells are mediated through the induction of apoptosis via both intrinsic and extrinsic pathways. Notably, treatment with the extract results in cell cycle arrest and an apoptotic cascade. These effects have been documented in vitro using various cancer cell lines, confirming the extract’s ability to trigger programmed cell death [112].

#### 2.2.9. Wound Healing

Chiu et al. (2012) [119] highlighted the possible mechanisms that explain the activity of PA, involving the following: (1) its analgesic ability demonstrated by two different analgesic test methods: acetic acid-induced writhing response and formalin tests; (2) its anti-inflammatory ability demonstrated by decreasing the swelling of carrageenan-induced mice paw edema and the levels of pro-inflammatory mediators (TNF-α and COX-2); (3) its antioxidant ability demonstrated by increased SOD and GRx levels and decreased MDA level (notably, PA does not increase the level of GPx as indomethacin does); and (4) possible mechanisms of its anti-inflammatory activities [119].

Kuo et al. (2012) [120] studied the healing activity of collection creams including *Plectranthus amboinicus* (Lour.) Spreng (Lamiaceae) and *Centella asiatica* (L.). Urb. (Umbelliferae) in 24 patients suffering from grade 3 foot ulcers. The patients were divided into two groups: one treated with an herbal cream and the other with hydrocolloid fiber dressings [120]. They compared the herbal cream with hydrocolloid fiber dressings. After 14 days of experiments, diabetic foot ulcers (DFUs) treated with the herbal cream showed a slight improvement, but this result was still better than that of the hydrocolloid fiber dressing [121].

Interestingly, the advancement of polyurethane foam dressing loading with the extract of *P. ambionicus* was intended for burn wound healing. The dressings were characterized in terms of their morphology, internal structure, and porosity and were evaluated for WVTR, absorption rate, and mechanical strength. Previous studies have indicated that a moisture vapor transmission rate (MVTR) between 2000 and 2500 g/m^2^/24 h is optimal for wound healing, as it maintains adequate moisture while minimizing the risk of desiccation [122]. All the formulations revealed a highly porous interior, and the porosity was more significant in formulations containing 1% of liquid paraffin. The porosity was further determined by the ethanol displacement method and the formulation with the highest porosity (FD4) was characterized by micro-CT. The MVTR and porosity tests are considered to be interdependent: the higher the porosity, the greater the loss of moisture from the wound milieu [122]. The suitability of dressings in handling wound exudate was determined by the absorption rate test. Formulations containing 1% liquid paraffin were found to possess higher absorptivity than the other formulations. Wound dressings should possess optimum tensile strength to ensure easy handling and application. The tensile test revealed that the dressings were flexible enough to withstand regular handling and could also conform to body movements without rupturing easily. Acute dermal irritation performed on rabbits showed no irritation, erythema, eschar, and edema. In vivo wound healing studies compared the healing efficacy of PAE foam dressings with the commercial formulation AgFix Foam™. The formulation with FD4 exhibited the best healing, which was confirmed by histopathological studies [49].

The diverse pharmacological effects of *P. amboinicus* are supported by its ability to modulate multiple molecular targets involved in inflammation, oxidative stress, wound healing, aging, and cancer progression (Table 3).

### 2.3. Cosmetic Formulations

Rich in bioactive compounds, *P. amboinicus* exhibits several pharmacological activities that make it a valuable ingredient in cosmetic formulations. In this part of the review, we explore the cosmetic potential of *P. amboinicus*, focusing on its use in traditional formulations, like ointments, but also as advanced nanoformulations.

The cosmetic potential of *P. amboinicus* is vast, owing to its rich phytochemical profile and diverse therapeutic properties. Its incorporation into various traditional formulations and advanced nanoformulations can lead to the development of innovative cosmetic products with enhanced efficacy and user benefits. Ongoing research and development in this area are likely to further uncover the extensive potential of this remarkable herb in skincare and personal care applications.

#### 2.3.1. Ointments and Creams

*P. amboinicus* is highly effective in ointments and creams due to its anti-inflammatory [42,63,88,112], antibacterial [42,63,88,112], and wound healing properties [49]. These topical formulations can treat skin conditions like eczema and minor wounds [124]. The essential oils extracted from *P. amboinicus* contain compounds such as carvacrol and thymol, which provide antiseptic and soothing effects [63]. This makes them ideal for skin applications aimed at reducing inflammation and promoting skin regeneration. Furthermore, the photoprotective potential demonstrated that *P. amboinicus* extracts make them suitable for use in the formulation of sunscreens [125].

#### 2.3.2. Lotions and Serums

Lotions and serums incorporating *P. amboinicus* extracts can benefit the skin by offering hydration and anti-aging effects [99]. The antioxidants present in the plant help combat oxidative stress, which can reduce the appearance of wrinkles and fine lines [22]. Furthermore, the terpenoids in *P. amboinicus* enhance skin barrier function, offering long-lasting hydration and protection against environmental damage [35].

#### 2.3.3. Hair Care Products

In hair care, *P. amboinicus* is utilized for its antimicrobial and anti-inflammatory properties, which are beneficial for treating dandruff and scalp irritations. Shampoos and conditioners containing *P. amboinicus* extracts can help maintain a healthy scalp, reduce dandruff, and promote hair growth by improving blood circulation to the scalp [126].

#### 2.3.4. Nanoformulations

Nanoformulations represent a cutting-edge approach in cosmetic science, offering enhanced stability, bioavailability, and efficacy of active compounds. The use of *P. amboinicus* in nanoformulations can significantly improve its performance in cosmetic products. In fact, several studies have demonstrated the involvement of these phytochemicals in the reduction and stabilization of different nanoparticles [47]. The use of biological methods to produce silver nanoparticles using *P. amboinicus* leaf extract was demonstrated to be simple, efficient, and eco-friendly [47].

*P. amboinicus* extracts can also be incorporated into liposomes to enhance their stability and control the release of active compounds. This makes them suitable for topical applications, improving the penetration and efficacy of the active ingredients [7].

The use of nanoemulsions enhance the solubility and absorption of hydrophobic compounds, ensuring better bioavailability and improved cosmetic benefits. These are fine oil-in-water or water-in-oil emulsions where the extracts of *P. amboinicus* are stabilized in nanosized droplets [80].

As for metallic nanoparticles, gold and silver nanoparticles can be synthesized using *P. amboinicus* extracts [47,66,71,127,128,129]. These metallic nanoparticles exhibit unique properties, such as enhanced antimicrobial activity, making them useful in formulations aimed at treating infections and promoting skin health. In this case, *P. amboinicus* extracts have been extensively used as a reduction agent for stabilization of metallic nanoparticles, such as Au [128], Ag [47], Zn [130], and Sn [131].

## 3. Materials and Methods

To achieve an in-depth understanding of *Plectranthus amboinicus*’ phytochemical composition and biological activity in dermatological contexts, a systematic review was conducted following the Preferred Reporting Items for Systematic Reviews and Meta-Analyses (PRISMA) strategy. Online databases, such as PubMed, Web of Science, and Scopus were inspected. A thorough exploration for full-text articles within these databases was conducted using two distinct sets of key search terms: “*Plectranthus amboinicus* & skin & chemistry” and “*Plectranthus amboinicus* & skin & dermocosmetic”. The initial research identified 298 articles. All retrieved publications were managed using Mendeley Reference Manager, and duplicates (*n* = 135) were ultimately removed. To ensure only relevant articles were included, specific selection criteria were applied during the initial screening: Firstly, review articles were not included. Next, only papers published between the years 2002 and 2024 qualified for the review. Finally, papers were only eligible if *Coleus amboinicus* and *Plectranthus amboinicus* were included.

## 4. Conclusions

The skin, our primary defense barrier, maintains overall health by protecting against environmental threats like UV radiation, injuries, and infections, while balancing moisture and lipids. Traditional herbal medicine has been pivotal in treating various ailments, with 80% of the population in developing countries relying on it. The *Plectranthus* genus, especially *P. amboinicus*, holds significant ethnobotanical value, offering antimicrobial, anti-inflammatory, and antioxidant properties. Studies have highlighted its therapeutic potential, supporting its traditional uses for treating skin diseases and other conditions.

The EOs from *P. amboinicus* exhibit a diverse range of chemical constituents, with carvacrol being the most common across various plant parts. The extracts and EOs demonstrate considerable antimicrobial activity against both Gram-positive and Gram-negative bacteria, as well as notable antifungal properties, particularly against dermatophytes. Additionally, *P. amboinicus* shows remarkable mosquito repellent and anti-parasitic effects, comparable to DEET, and potent anti-inflammatory properties by inhibiting pro-inflammatory cytokines.

The plant’s rich polyphenolic content contributes to its significant antioxidant properties, preventing conditions like hyperpigmentation and premature aging. *P. amboinicus* also exhibits cytotoxic activity against various cancer cell lines and promotes wound healing through its analgesic, anti-inflammatory, and antioxidant abilities. The comprehensive exploration of *P. amboinicus* underscores its diverse therapeutic potential across infectious diseases, oncology, and wound care. Further research and clinical trials are warranted to fully elucidate its mechanisms of action and optimize its therapeutic applications, paving the way for its integration into mainstream medical practices.

## Figures and Tables

**Table 1:** Phytochemicals identified in the essential oil of *P. amboinicus* (EO-Pa) extracted from stems, whole plants, or aerial parts. The plant part used for extraction is indicated in parentheses. Values represent the percentage or area under the peak (%).

Compound	Petrolina, Brazil (Stems) [46]	Cariré, Brazil (Aerial) [37]	Havana, Cuba (Aerial) [74]	Mauritiu, Madagascar (Whole) [77]	Uttarakhand, India (Aerial) [86]	Taiwan (Aerial) [81]	Cairo, Egypt (Dried Herb) [84]	Cairo, Egypt (Whole) [83]		Number of Results
Alloaromadendrene							5.21			1
α-Bergamotene								2.96	2.56	2
α-cis-Bergamotene		0.84								1
(*E*)-α-Bergamotene			2.7	4.0						2
β-bisabolene				0.3					0.87	2
Borneol								0.24	0.4	2
β-Bourbenene							2.12			1
α-Cadinol				0.4	1.6		5.67			3
δ-Cadinol							6.44			1
Calamenene				0.2						1
Camphene				0.8	0.3					2
Camphor				12.9						1
γ-Cadinene							0.35			1
δ-Cadinene				1.4			6.02			2
δ-3-Carene				15.2						1
Carvacrol	76.33	88.17	71	17.9	0.45	61.53	5.96	2.96	3.11	9
Caryophyllene	9.33									1
(*E*)-Caryophyllene					2.02					1
β-Caryophyllene			4.2	6.1		12.79	0.71	11.6	14.98	6
Caryophyllene oxide		5.85	1.6	0.9	1.24	0.37	0.48			6
14-Hydroxy-(*Z*)-caryophyllene			0.2							1
1.8-Cineole		2.01		0.2						2
α-Copaene				1.1						1
β-Copaene							0.39			1
ρ-Cymene	5.35	0.72	9.7	9.9		9.42	2.55	10.68	13.65	8
α-Fenchone				0.2						1
α-Humulene			1.3	1.6		3.24		1.3	0.35	5
Humulene epoxide II			0.3							1
trans-Isocarveol						0.51				1
Transcycloisolongifol-5-ol							1.11			1
Limonene				1.2			8.03			2
Linalool				0.3	0.49					2
epi-α-Muurolol							0.69			1
T-Muurolol				0.3						1
α-Muulorene							7.31			1
γ-Muulorene							5.51			1
α-Muulorene-4-hydroxy							1.17			1
Myrcene			0.6	1.2		0.53		2.1	2.45	5
Myristicin				0.2						1
(*E*)-β-ocimene							0.57			1
Oct-1-en-3-ol			0.6	0.6	1.17			3.25	2.37	5
Oplupanone							0.98			1
α-Phellandrene				0.8		0.2				2
β-Phellandrene				0.5						1
α-Pinene				0.7		0.22				2
β-Pinene							0.98	0.68	1.1	3
cis-Sabinene hydrate					0.42					1
α-Selinene				0.7						1
β-Selinene				4.2			0.29			2
Spathulenol					0.24					1
Terpinen-4-ol			1.4		1.03		2.93	0.93		4
Terpinene	8.99									1
α-Terpinene			0.6	4.1		1.73	0.22	3.15	1.87	6
γ-Terpinene		0.45	4.3	6.6		8.51	8.64	18.86	15.89	7
α-Terpineol				0.2						1
Terpinolene				1			0.63	0.93	0.93	4
Tetradecene					0.23					1
Thymol				0.3	83.39	0.21		32.42	34.89	5
Thymol acetate					0.46			1.43	0.9	3
α-Thujene			0.8	0.6		0.41		0.7	0.46	5

The number of times a compound was reported across the selected studies was also included to illustrate how common certain compounds are in the EO-Pa. Compounds that represented less than 0.2% of the oil were not included. For the report by Sabra et al. (2018) [83], only samples from February (4-day irrigation period) and August (16-day irrigation period) were included. The parts of the plant used for extraction are in parentheses. Cell background color in the “Number of Results” column reflects the frequency of detection across studies: Green = Frequently reported (≥5 times, darker green corresponds to more occurrences); Yellow = Moderately reported (2–4 times); Red = Rarely reported (1 time).

**Table 2:** Phytochemicals identified in the essential oil of *P. amboinicus* (EO-Pa) extracted from leaves. The collection region is indicated for each sample. Values represent the percentage or area under the peak (%). The number of studies reporting each compound is also included to indicate how commonly each compound appears in EO-Pa. Compounds contributing less than 0.2% were excluded.

Compound	Malaysia [92]	Cambodia [90]	Mangudi, India [89]	Rajahmundry, India [48]	Mysore, India [31]	Oshida, India [88]	Punjab, India [58]	Tiruchirappalli, India [79]	Fortaleza, Brazil [21]			Nova Odessa, Brazil [87]	Alegre, Brazil [82]	Apiacá, Brazil [17]	Paraíba, Brazil [80]	Ceará, Brazil [78]	Number of Results
Cyclopropa [5,6]-A-nor-5,alpha,-androstane-3,7-dione, 3′,6,beta,-dihydro-17,beta,-hydroxy-3′,3′-dimethyl-, acetate							0.21										1
2Z-octenol acetate			0.96														1
Phthalic acid															4.32		1
Alloaromadendrene							0.21										1
Allopregnanolone							0.27										1
Aromadendrede epoxide	0.3					0.5											2
Aromadendrede oxide								11.19									1
Isoaromadendrene epoxide						0.5											1
Adamantane							0.2										1
β-Bisabolene	1.8							5.77									2
α-Bergamotene					3.9				1.78								2
α-cis-Bergamotene	7.7																1
(E)-α-Bergamotene	0.3	2.5										8.19					3
γ-Bergamotene															3.16		1
Borneol			0.26														1
Bornyl isovalerate							2.47										1
2C-E							2.7										1
δ-Cadinene					0.9												1
Carene						15.89											1
4-Carene						0.56											1
Carvacrol	54.4	65.2	29.25	1.2	70	20.25						37.7	88.61		33.5		9
Carvacrol acetate	0.2					1.07											2
Dihydro carvel			0.23														1
Caryophyllene							0.27	19.34									2
α-Caryophyllene					6.2												1
β-Caryophyllene	8.9	5			1.9				3.09							2.8	5
(Z)-Caryophyllenne												14.07	2.39				2
(E)-Caryophyllene															4.63		1
Isocaryophyllene							12.18										1
Caryophyllene oxide	6	1.7	5.83		1	5.76	5.05	10.78	1.36		0.2				0.62		10
β-Cedrene epoxide	0.3																1
1,8-Cineole				0.8													1
1-epi-Cubenol			0														1
n-tetraContane							0.33										1
Octatriacontyl pentafluoropropionate							1.48										1
α-Copaene							1.03										1
α-Cubebene					0.8												1
Cuminol							18.57										1
o-Cymene						19.41											1
ρ-Cymene		8.5	6.51	0.3							1.65	12.01		19.46	28.2	10.3	8
3-Methylheptadecane							0.02										1
Eicosahydrodibenzo(a,i)fluorene							1.22										1
Elixene							2.9										1
Eremophilene						0.44											1
Eudesma-4, 11-diene					1.8												1
Eugenol													1.59				1
(Z)-α-farnesene	1.4																1
(E)-β-farnesene	0.2						1.55					0.39					3
Globulol							1.41										1
1,3,3-Trimethyl-2-vinyl-1-cyclohexene							0.21										1
α-Himachalene						0.44											1
α-Humulene	3.1	1.5	9.67									3.83			1.17		5
Humelene oxide								3.68									1
Humulene epoxide II	1.1																1
Irone							0.27										1
α-Isomethyl ionone							0.86										1
Ledol							0.62										1
Limonene												0.46			0.84		2
D-Limonene							0.82										1
Linalool							0.66										1
Linolenic acid, methyl ester							0.83										1
epi-Longipinanol	1.1																1
p-Menthatriene							4.45										1
Methyl chavicol			0.28														1
Methyl octanoate			0.42														1
β-Myrcene		0.7			0.4	0.33	6.17					0.97		12.59	2.03		7
Myrcene dissulfide						0.33											1
Nopinene						0.33											1
Oct-1-en-3-ol		0.3															1
(E)-β-Ocimene							64										1
cis-β-Ocimene							0.39										1
1-octene			0														1
Palmitic acid							1.31	3.54									2
2-Cyclopentylcyclopentan-1-one							0.24										1
2,3,4,5-Tetramethylcyclopent-2-en-1-ol							0.48										1
Perillen						1.12											1
4-tert-Butyl-2-(5-tert-butyl-2-hydroxyphenyl)phenol							0.32										1
Phytol								3.73									1
Pichtosin							0.2										1
α-Pinene							0.2					0.24			0.38		3
β-Pinene						0.26											1
(-)- β Pinene							0.2										1
α-Phellandrene		0.2													0.31		2
β-Phellandrene		0.4															1
β-Sesquiphellandrene	0.4																1
Sabinene						0.26											1
trans-Sabinene hydrate			0.22														1
Selinene								10.81									1
β-Selinene			2.01														1
δ-Selinene	1.7																1
γ-Selinene							2.55										1
Seline-4(14),11-diene								6.72									1
Shyobunol							0.65										1
Spathulenol				0.2													1
Stearic acid							0.36										1
Terpinen-4-ol	0.2			0.2	1.2		1.65		90.55	95.39	98.03	1.39					8
L-4-Terpineol															1.52		1
α-Terpinene		1.7	0.61									1.96			2.52		4
γ-Terpinene		10	7.76		5.3	0.56	2.46					14.74			14.77	9.9	8
α-Terpineol		1.1	3.28				0.22										3
α-Terpinolene							7.18							9.86			2
4-Thujanol							0.24										1
α-Thujene		0.2													0.83		2
2-phenyl ethyl tiglate			1.38														1
Thymol		0.5	21.66	94.3	0.3	20.17						0.52		45.64		64.3	8
Thymol acetate	0.2																1
Undecanal			8.29														1
Valeranone							6.47										1
Verbenol	1.6																1
Vinyl amyl carbinol							0.39								0.74		2
Cycloheptane,4-methylene-1-methyl-2-(2-methyl-1-propen-1-yl)-1-vinyl								4.75									1
Viridiflorol							0.62										1
Widdrol								1.56									1

Cell background color in the “Number of Results” column reflects the frequency of detection across studies: Green = Frequently reported (≥5 times, darker green corresponds to more occurrences); Yellow = Moderately reported (2–4 times); Red = Rarely reported (1 time).

**Table 3:** Molecular targets regulated by *P. amboinicus* and their associated biological activities.

Molecular Target	Biological Activity	Mechanism/Role	References
Tyrosinase	Anti-aging, skin-lightening	Inhibition reduces melanin synthesis and hyperpigmentation	[99,123]
Collagenase	Anti-aging, anti-wrinkle	Inhibition protects extracellular matrix and prevents skin degradation	[99,123]
TNF-α (Tumor Necrosis Factor-alpha)	Anti-inflammatory	Downregulation reduces systemic and local inflammation	[119]
IL-8 (Interleukin-8)	Anti-inflammatory	Inhibition reduces inflammatory cell recruitment	[55]
NF-κB (Nuclear Factor kappa B)	Anti-inflammatory	Inhibition suppresses transcription of pro-inflammatory cytokines	[55]
COX-2 (Cyclooxygenase-2)	Anti-inflammatory, wound healing	Downregulation contributes to pain relief and inflammation control	[119]
SOD (Superoxide Dismutase)	Antioxidant	Enhances antioxidant defense by neutralizing superoxide radicals	[119]
GRx (Glutathione Reductase)	Antioxidant	Maintains glutathione homeostasis and redox balance	[119]
MDA (Malondialdehyde)	Antioxidant, anti-aging	Reduced levels indicate protection from lipid peroxidation	[119]
Ergosterol synthesis	Antifungal	Disruption impairs fungal membrane integrity	[31]
Cancer cell viability pathways (e.g., p53, Bcl-2 inferred)	Anticancer	Induction of cytotoxicity and apoptosis in cancer cells	[58,59,112]

## Data Availability

No new data were created or analyzed in this study. Data sharing is not applicable.
